# Assessment of the Mechanical and Corrosion Properties of Mg-1Zn-0.6Ca/Diamond Nanocomposites for Biomedical Applications

**DOI:** 10.3390/nano12244399

**Published:** 2022-12-09

**Authors:** Hüseyin Şevik, Selma Özarslan, Hajo Dieringa

**Affiliations:** 1 Department of Metallurgical and Materials Engineering, Faculty of Engineering, Mersin University, Mersin 33343, Turkey; 2 Department of Physics, Science and Art Faculty, Hatay Mustafa Kemal University, Antakya 31034, Turkey; 3 Institute of Material and Process Design, Helmholtz-Zentrum Hereon, Max-Planck-Str. 1, 21502 Geesthacht, Germany

**Keywords:** Mg-Zn-Ca/diamond composite, diamond nanoparticles, mechanical properties, corrosion resistance

## Abstract

In this work, the microstructure, mechanical properties, and corrosion behavior of the Mg-1Zn-0.6Ca matrix alloy (ZX10), reinforced by adding various amounts of nanodiamond particles (0.5, 1, and 2 wt.%), prepared by the ultrasound-assisted stir-casting method, were investigated as they are deemed as potential implant materials in biomedical applications. Microstructure, nanoindentation, mechanical tensile, immersion, and potentiodynamic polarization tests were performed for evaluating the influence of the addition of nanodiamond particles on the alloy’s mechanical and biocorrosion properties. The results revealed that the addition of nanodiamond particles causes a reduction in the alloy’s grain size. The alloy’s nanohardness and elastic modulus values increased when the amount of added nanodiamond particles were increased. The nanocomposite with an addition of 0.5% ND showed the best composition with regard to an acceptable corrosion rate as the corrosion rates are too high with higher additions of 1 or 2% NDs. At the same time, the yield strength, tensile strength, and elongation improved slightly compared to the matrix alloy.

## 1. Introduction

Recently, the improvement of biodegradable implants has attracted great attention as an alternative for eliminating the long-term complications induced by permanent implants intended for cardiovascular and orthopedic applications [1,2,3,4,5]. Magnesium is one of the most promising elements for use in biodegradable implant applications due to its biocompatibility, and the density and Young’s modulus are comparable with bone [6,7,8,9,10,11]. However, pure magnesium and its alloys are naturally biodegradable due to corrosion occurring when they are placed inside the body, and this causes the early decline of mechanical properties and implant failure; in other words, the insufficient strength and weak corrosion resistance of Mg alloys still prevent their widespread use [6,12,13,14,15]. For this reason, finding out the best way of obtaining biocompatible magnesium alloys, having both acceptable mechanical properties and corrosion resistance, has been among the major topics in recent years. Many studies are being performed by scientists in order to overcome the abovementioned problems, and it can be stated that the main topics of these studies are the development of novel biocompatible Mg alloys by the addition of Ca [16], Zn [15,17], Sr [18], Sn [19,20,21], and rare earth elements (RE) [1,8,22]; the production of nanocomposites by the addition of various ceramic-reinforcing materials into these alloys such as SiC [23], carbon nanotubes [24,25], hydroxyapatite [26], tricalcium phosphate [27], Y_2_O_3_ [28], ZnO [29], and Al_2_O_3_ [30]; and the development of protective coatings such as microarc oxidation [31,32,33,34,35].

Among the novel biocompatible Mg alloys, the Mg-Zn-Ca ternary alloy is a very appealing combination due to its tolerable high daily intake for human body [16]. Therefore, various combinations of the Mg-Zn-Ca ternary alloy are being studied by scientists. They report that the Mg-Zn-Ca ternary alloy basically contains primary α-Mg grains, and Ca_2_Mg_6_Zn_3_ and Mg_2_Ca intermetallic phases. The corrosion of the Mg-Zn-Ca alloy can show differences based on the presence of these intermetallic phases arising from the atomic content ratio of zinc to calcium. It has been reported that if the atomic content ratio of Zn/Ca is above 1.0–1.2, then it will just form the Ca_2_Mg_6_Zn_3_ intermetallic phase, and if its atomic ratio is below this range, then it will form both the Mg_2_Ca and Ca_2_Mg_6_Zn_3_ intermetallic phases [36,37,38]. While the Mg_2_Ca intermetallic phase is detrimental for the corrosion resistance of Mg, the Ca_2_Mg_6_Zn_3_ intermetallic phase ensures a barrier against corrosion because the electrochemical potentials are Mg_2_Ca < α-Mg < Ca_2_Mg_6_Zn_3_ [39,40,41].

Nanocomposite is a multiphase solid material in which at least one of the phases (typically reinforcement) is in the nanometer range [23]. Previous studies have shown that the nm scale reinforcement is remarkable at reducing the grain size of the Mg alloys and thus at improving the mechanical properties via the grain refinement (strengthening) mechanism of Hall–Petch [42]. In addition, this mechanism may enhance the corrosion resistance of the Mg alloys as numerous scientists have proved in their studies [7,29,30,43]. For example, Lin et al. [44] have reported that the mechanical properties and corrosion resistance of the as-extruded Mg-Zn-Ca alloy are enhanced by the addition of MgO nanoparticles by the use of high shear stirring. The enhancement of both the mechanical properties and corrosion resistance of the alloy is primarily ascribed to grain refinement, and good interfacial bonding between the matrix and MgO nanoparticles. Nano-diamond (ND) is considered as the most biocompatible material among all known carbon derived nanomaterials. Recent studies have revealed that ND could be used as a new corrosion inhibiting material for Mg alloys. For instance, a study performed by Gong et al. [45] produced the Mg/nanodiamond composites by the use of the powder metallurgy technique. They noticed that nanodiamond particles play an effective role in the formation of the passive calcium phosphate layer on the surface, and they stated that the corrosion resistance of pure Mg is improved by the addition of nanodiamond particles. On the other hand, the detrimental effect of nano carbon on the corrosion resistance of Mg because of microgalvanic action among the α-Mg matrix and nano carbon has been reported by some studies [24,46,47].

In the present study, it was intended to produce biocompatible Mg-Zn-Ca-based nanocomposites through the addition of nanodiamond (ND) particles of various percentages by the use of the ultrasound-assisted gravity casting method. The sonication of the melt with the particles introduced breaks up particle agglomerates through cavitation and acoustic streaming. The resulting homogeneously distributed nanodiamonds can more effectively unfold their effect on the properties of the nanocomposite material. The effect of nanodiamond (ND) particles on the properties of the Mg-1Zn-0.6Ca-based alloy (ZX10) was investigated by the use of metallurgical analysis techniques.

## 2. Materials and Methods

The Mg-1Zn-0.6Ca alloy was chosen as the matrix alloy for the nanocomposites. Pure magnesium ingot, high-purity zinc (99.9%), and calcium (99.9%) granules were used as the raw materials of the desired matrix alloy, whose composition is listed in Table 1. Nanodiamond particles (160 nm diameter, +15 mV Zeta potential, and 1.7% ash), produced by Adamas Nano (Raleigh, North Carolina, USA), were used as reinforcement. Pure Mg ingot was melted in a steel crucible by the use of an electrical resistance furnace, and then pure calcium and zinc were added and kept under a protective atmosphere of Ar gas containing 4 vol.% SF_6_ for 20 min. at 740 °C casting temperature. After the preparation of the matrix alloy, nanodiamond particles were added and then ultrasonic agitation for 5 min was used to ensure the homogeneous dispersion of the nano diamond particles in the matrix by the use of the ultrasound-assisted stir casting method (UP200st, Hielscher, Germany, 0.15 kW and by the use of a titanium sonotrode). After stirring, the solidification was carried out in a water bath. When the temperature of the alloy dropped to room temperature, the alloy was then removed from the crucible. All of the as-cast materials obtained were named as, according to ASTM, ZX10, ZX10-0.5ND, ZX10-1ND, and ZX10-2ND.

The metallographic specimens were prepared through conventional grinding with silicon carbide abrasive paper (220–2500 grit), fine polishing with 1 μm colloidal silica (OPS) and 0.25 μm diamond suspension, and etching in an acetic picric solution (5 mL acetic acid, 8 g picric acid, 10 mL distilled water, and 100 mL ethanol). Microstructural studies were performed through optical microscopy (OM; Olympus BX51) and scanning electron microscopy (SEM; TESCAN VEGA3) by the use of the Tescan energy-dispersive X-ray (EDX) spectrometer. The average grain size of the matrix and of the nanocomposites was calculated by the use of AnalySIS Pro software FIVE (Olympus Soft Imaging Solutions, Germany). Line segments were drawn on the optical microstructure. The number of grain boundaries intercepted with the line was counted. The average grain size was calculated as the ratio of the number of intersections to the length. To collect the quantitative grain size data, at least 50 random counts were measured in the optical microstructure. The phase identifications were proven through X-ray diffraction (XRD) by the use of a Cu K_α_ radiation (wave length: 0.15418 nm) at 40 kV and 40 mA.

Nanoindentation tests were performed in order to evaluate the change in the hardness and elastic modulus of the matrix following the addition of nanodiamond particles. Before performing the indentations, the surfaces of the samples were fine polished. Nanoindentation tests were performed on a nanoindenter (Hysitron TI950 TriboIndenter) by the use of a standard Berkovich diamond indenter tip with a tip radius of 100 nm, calibrated with a fused quartz reference sample, as well as by the use of a scanning probe microscope (SPM) for recording the morphology of indents (images: 10 × 10 µm^2^). The test consisted of a series of six indents arranged in a three-by-two pattern with 10 μm spacing between indents performed under load control. Six grits were applied to each sample with a force of 5 mN. However, the load-displacement curves closest to the mean value are given in the results. The holding time is 2 s. 

The tensile samples were tested as per the ASTM E8M-03 standard in a universal testing machine (Zwick 050 machine (Zwick GmbH and Co. KG, Ulm, Germany)) at a body temperature of 36.5 °C and at a ram speed of 0.2 mm/min. Each material was tested five times.

The corrosion properties of the alloy and of the nanocomposites were investigated by immersion tests and potentiodynamic polarization tests in Hank’s Balanced Salt Solution (HBSS: 8 g/L NaCl, 0.4 g/L KCl, 0.4 g/L MgCl_2_·6H_2_O, 0.35 g/L NaHCO_3_, 0.25 g/L NaH_2_PO_4_·2H_2_O, 0.06 g/L Na_2_HPO_4_·2H_2_O, 0.19 g/L CaCl_2_·2H_2_O, 0.06 g/L MgSO_4_·7H_2_O, and 1 g/L glucose) at body temperature (36.5 °C). Before the immersion test, each sample surface, having a length of 15 mm on each side, was polished, and cleaned with ethanol. The initial pH values of the solutions were set as 7.4. After the immersion test of 384 h, oxides, which had been formed on the corroded samples, were cleaned with 200 g L^−1^ CrO_3_ solution, and then the loss of mass was measured by the use of a digital analytical balance with an accuracy of 0.0001 g. In addition, the pH changes of the solution were observed at 1, 2, 4, 8, 12, 24, 48, 96, 192, and 384 h.

Potentiodynamic polarization tests were performed by the use of a CHI602E model potentiostat–galvanostat device (CHI Instruments Inc., Shanghai, China) at body temperature (36.5 °C) in 100 mL HBSS through a conventional three-electrode set-up (a platinum plate as the counter electrode, a saturated Ag-AgCl electrode as the reference, and the sample as the working electrode). The exposed surface area of the samples was about 1 cm^2^. The potentiodynamic polarization test was conducted at a potential scanning speed of 1 mV/s. In order to obtain a steady-state corrosion potential (E_corr_), the samples were immersed in the solution for about 1 h prior to measurements. Polarization parameters were provided by the use of the cathodic Tafel extrapolation technique. In order to ensure well reproducibility of data, all samples were handled for three times.

## 3. Results

### 3.1. Microstructure and Characterization

**Figure 1:**
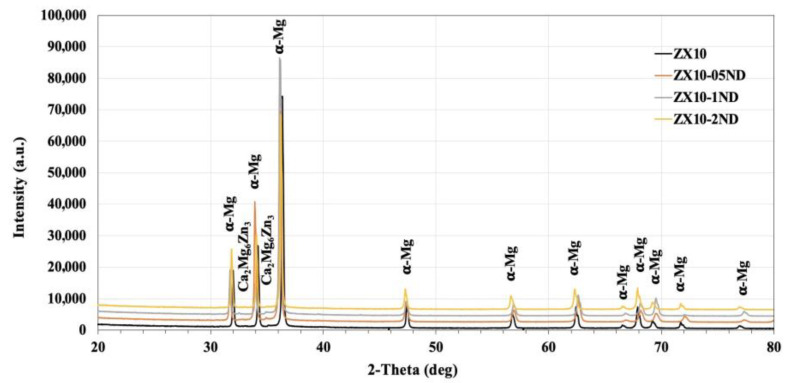
The X-ray diffraction analysis patterns of the alloy and nanocomposites.

**Figure 2:**
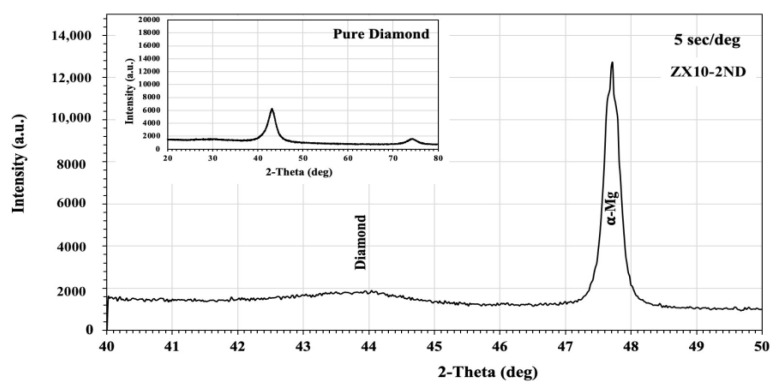
The X-ray diffraction analysis pattern of the ZX10-2ND.

Figure 1 demonstrates the XRD patterns of the ZX10 alloy and of the ZX10-0.5ND, ZX10-1ND, and ZX10-2ND nanocomposites. As it was predicted, the most intense peaks obtained from the ZX alloy and nanocomposites corresponded to the α-Mg phase, while the peaks with lower intensity corresponded to the Ca_2_Mg_6_Zn_3_ secondary phase. On the other hand, the peaks obtained from the XRD patterns of nanocomposites did not coincide with any diamond peaks. Therefore, the XRD analysis for the ZX10-2ND was repeated at 40°–50° (2θ), and at 5 sec/deg (Figure 2) the peak with lower intensity was detected at 43°, which coincided with that of the diamond peak.

**Figure 3:**
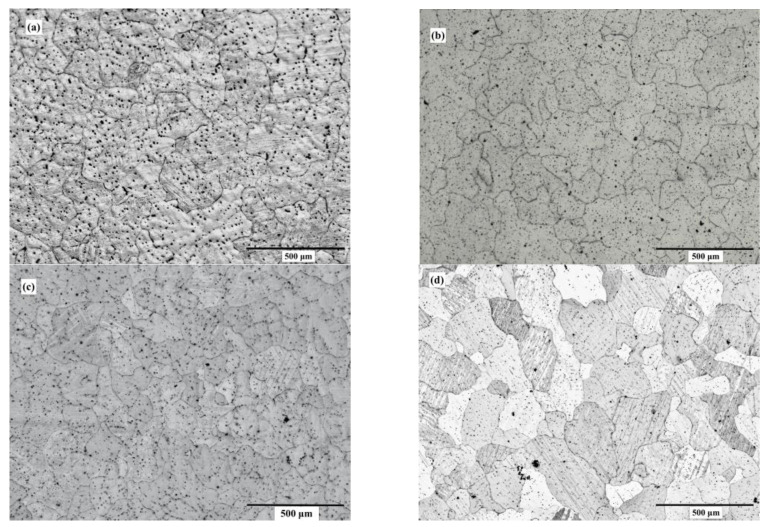
Optical microstructures of (**a**) ZX10 alloy, (**b**) ZX10-0.5ND, (**c**) ZX10-1ND, and (**d**) ZX10-2ND.

**Figure 4:**
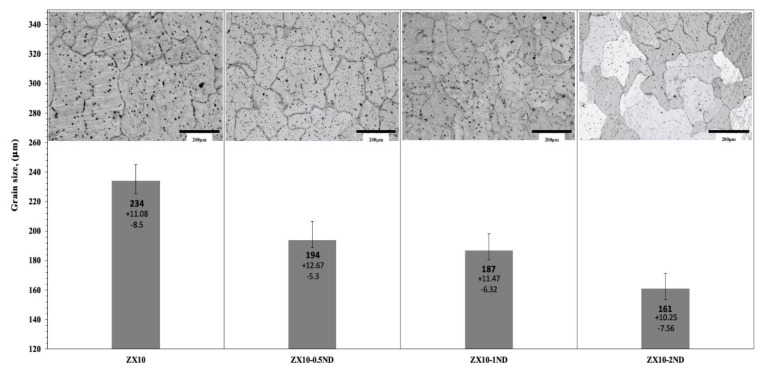
The grain size of the alloy and nanocomposites.

Figure 3 and Figure 4 depict the optical microstructures and grain size of the ZX10 alloy, and of the ZX10-0.5ND, ZX10-1ND, and ZX10-2ND nanocomposites, respectively. As it can be seen in Figure 3 and Figure 4, the average grain size of the ZX10 was reduced by increasing the amount of added nanodiamond particles. The average grain size of the ZX10 nanocomposite was found to be 234 µm + 11.08/−8.5 µm (the full range of the measured grain size). The average grain size was decreased from 234 µm + 11.08/−8.5 µm to 194 µm + 12.67/−5.3 µm, 187 µm + 11.47/−6.32 µm, and 161 µm + 10.25/−7.56 µm, respectively, by the addition of nanodiamond particles. This grain refinement was attributed to the refining efficiency of nanodiamond particles that can probably act as potent nucleation sites for α-Mg during solidification. Furthermore, Figure 3 reveals that the second phases in the alloy and nanocomposites are mainly present along the grain boundary and within the grain.

**Figure 5:**
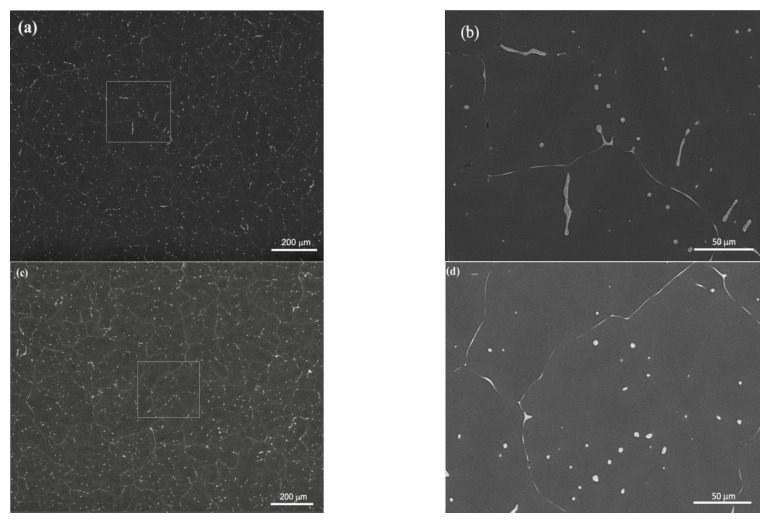

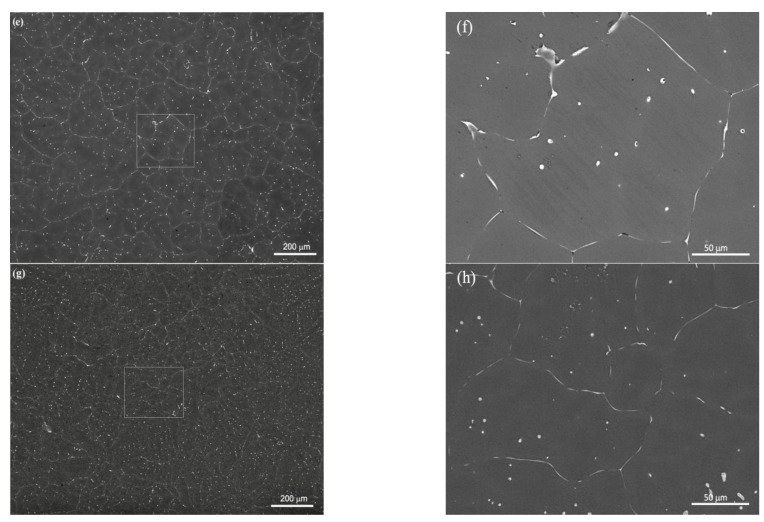
SEM microstructures of (**a**,**b**) ZX10 alloy, (**c**,**d**) ZX10-0.5ND, (**e**,**f**) ZX10-1ND, and (**g**,**h**) ZX10-2ND. (**b**,**d**,**f**,**h**) are higher magnifications of boxed zones in (**a**,**c**,**e**,**g**).

**Figure 6:**
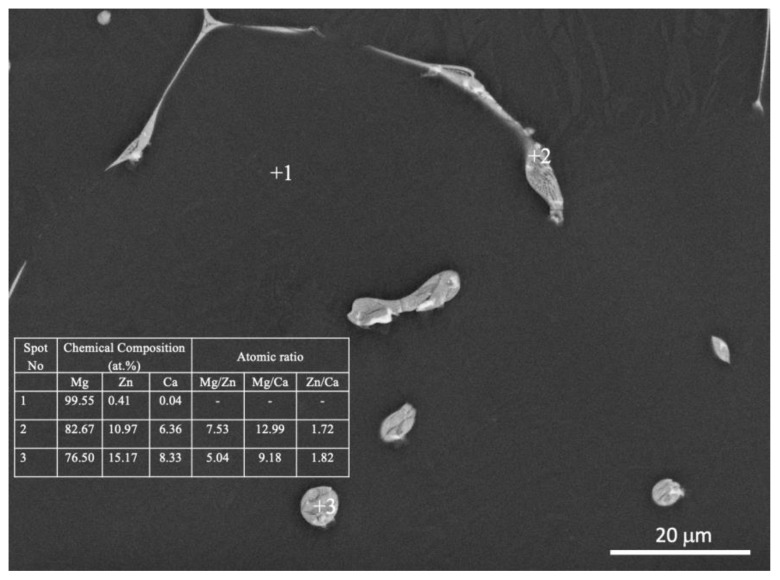
EDS analysis of the ZX10 alloy.

**Figure 7:**
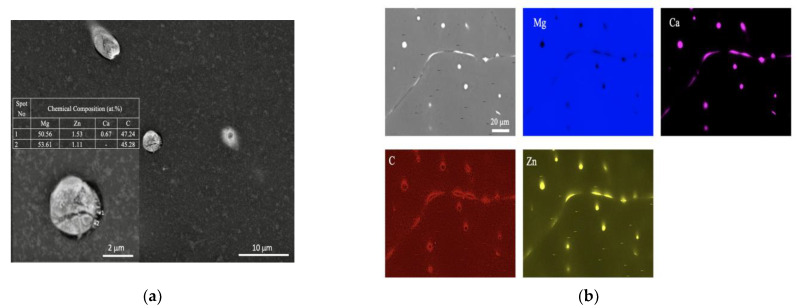
EDS analysis of the ZX10-2ND nanocomposite. (**a**) SEM micrograph of ZX10-2ND with magnification of the intermetallic particle shown in the middle including EDS analysis on two points and (**b**) EDS elemental mapping of Mg, Ca, C, and Zn performed on the left micrograph in the upper row.

SEM, applied at different magnifications of the ZX10 alloy, and of the ZX10-0.5ND, ZX10-1ND, and ZX10-2ND nanocomposites, was performed in order to better understand the details of morphology of the second phases, and the distribution of nanoparticles, and it is shown in Figure 5. It can clearly be seen from Figure 5a,b that the ZX10 alloy consists of primary α-Mg, and the second phases are semi-continuously distributed both on the grain boundaries and within the grain in the form of a spherical and large island-like (arrow) morphology. In addition, when Figure 5a,c,e,g are considered, it can be said that the formation of the large island-like morphology decreased by the addition of nanodiamond particles. Furthermore, it can be observed in Figure 5h that some agglomerations of nanodiamond particles occurred within the grains. In the Mg-Zn-Ca ternary alloy, the second phase is identified as the eutectic phase, and it consists of (α-Mg + Ca_2_Mg+ Ca_2_Mg_6_Zn_3_) or (α-Mg + Ca_2_Mg_6_Zn_3_) depending on Zn/Ca atomic ratio as described above [37,38,39]. In the present study, the chemical composition of the primary α-Mg, and the second phase in the ZX10 alloy, were determined by the use of EDS analysis as shown in Figure 6. The EDS results revealed that a small amount of zinc is dissolved in the primary α-Mg grain (Spot 1). Furthermore, the atomic ratios of Zn/Ca at spots 2 and 3 were 1.72 and 1.82, respectively. Therefore, the intermetallic phase is identified as Ca_2_Mg_6_Zn_3_. Figure 7 provides more detail on the locations of nanodiamond particles within the nanocomposites. It can be seen from Figure 7a that some of the nanodiamond particles were located around the intermetallic. The inset in the lower left of Figure 7a represents a magnification of the intermetallic particle shown in the middle of Figure 7a. The nanodiamond particles possibly interacted with the Ca^2+^ ions during solidification; therefore, the nucleation of the second phase started on the surfaces of the nanodiamonds, and in addition, it may have prevented its growth as the nanodiamond particles were positioned around the second phase. Figure 7b represents an EDS elemental mapping of Mg, Ca, C, and Zn performed on the left micrograph in the upper row.

### 3.2. Mechanical Test

**Figure 8:**
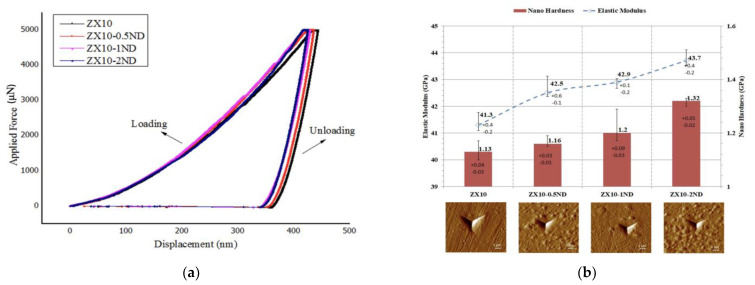
(**a**) Load-displacement curves of the ZX10 alloy and ZX10-0.5ND, ZX10-1ND, and ZX10-2ND nanocomposites; (**b**) nano-hardness values and reduced elastic modulus of the ZX10 alloy and ZX10-0.5ND, ZX10-1ND, and ZX10-2ND nanocomposites with scanning probe microscopy (SPM) images (10 × 10 µm^2^) after a nanoindentation test.

Figure 8a illustrates the load-displacement curves for the alloy and for the nanocomposites. After indentation, the reduced elastic modulus and nanohardness of the alloy and of the nanocomposites were calculated by the use of the procedure specified by Oliver and Pharr [48], and they are shown with scanning probe microscopy (SPM) images in Figure 8b.

As it was revealed by Figure 8a,b, the hardness and elastic modulus values of the ZX10 alloy were found to be 1.13 and 41.31 GPa, respectively. When the ZX10 alloy was considered, the hardness and elastic modulus values of the alloy were increased by the addition of nanodiamonds, and almost 3%, 6%, and 17% increases occurred in the hardness values for the ZX10-0.5ND, ZX10-1ND, and ZX10-2ND nanocomposites, respectively. Regarding the elastic modulus value, the increase was obtained approximately by 3%, 4%, and 6%. In addition, no pop-in and fractures were observed on the surface after indentation through the curves (Figure 8a) or through the SPM images. The main reasons for the improvement in the alloy’s hardness and elastic modulus were the smaller grain size, and the dispersion hardening due to the hard nanodiamond particles.

**Figure 9:**
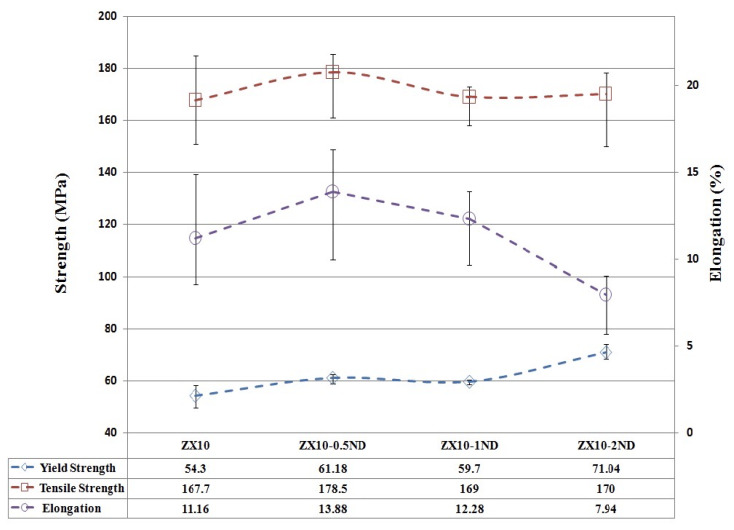
The yield strength, tensile strength, and elongation graphs of ZX10 alloy and nanocomposites at body temperature. Error bars represent full range of measured values.

Figure 9 shows the yield strength, tensile strength, and elongation value of the ZX10 alloy and nanocomposites at a body temperature of 36.5 °C. It can be seen from Figure 9 that the yield strength, tensile strength, and elongation values of the ZX10 alloy were measured as 54.3 MPa, 167.7 MPa, and 11.16%, respectively. The yield strength of the ZX10 alloy was improved with increasing nanodiamond particle content. The yield strength of ZX10-0.5ND and ZX10-1ND was slightly higher than that of ZX10 on the same level. ZX10-2ND showed the highest yield strength. However, the ZX10-0.5ND nanocomposite exhibited the best tensile strength value (178.5 MPa) and elongation value (13.88%) within the measurement uncertainty. It was observed that when more nano diamond was added, both the tensile strength and the elongation value decreased. It is known that the addition of nano diamond particles is beneficial in reducing the grain size, which can be considered as the main reason for the enhancement of mechanical properties. Another reason for the increase can be the dispersion strengthening effect (the Orowan strengthening mechanism) of hard nano diamond particles in the nanocomposite, which serves as a barrier to motion of dislocations. The decrement in ductility and tensile strength can be attributed to the micro-voids that formed between the matrix and the nano diamond clusters, the weak binding force of the nano-particles around those clusters (Figure 5), and the propagating of the cracks during the tensile testing.

### 3.3. Immersion Test

The anodic dissolution of α-Mg (reaction (1)) and the cathodic decomposition of water with the evolution of hydrogen (reaction (2)) mainly accompany the corrosion of magnesium. The production of OH^−^ ions causes an increase in the pH value at the corrosion interface, and then due to the rise in the pH value (pH > 9), magnesium hydroxide film forms (reaction (3)). In addition to this, the Mg(OH)_2_ layer deposits on the surface, but this layer does not effectively protect magnesium, especially in solutions containing Cl^−^ ions such as HBSS because the Mg(OH)_2_ layer is not compact [49,50]. The Mg (OH)_2_ layer reacts with the Cl^−^ ions and causes reaction 4, resulting in the increase in the pH value of solution. All of these circumstances, such as the change in pH value or film formation, are often used by researchers for evaluating the corrosion behavior of magnesium [21,44,48,49,50].

Mg → Mg^2+^ + 2e^−^ (anodic reaction) (1)

2H_2_O + 2e^−^ → H_2_ (g) + 2OH^−^ (cathodic reaction) (2)

Mg^2+^ + 2OH^−^ → Mg (OH)_2_ (product formation) (3)

Mg (OH)_2_ + 2Cl^−^ → MgCl_2_ +2OH^−^ (4)

**Figure 10:**
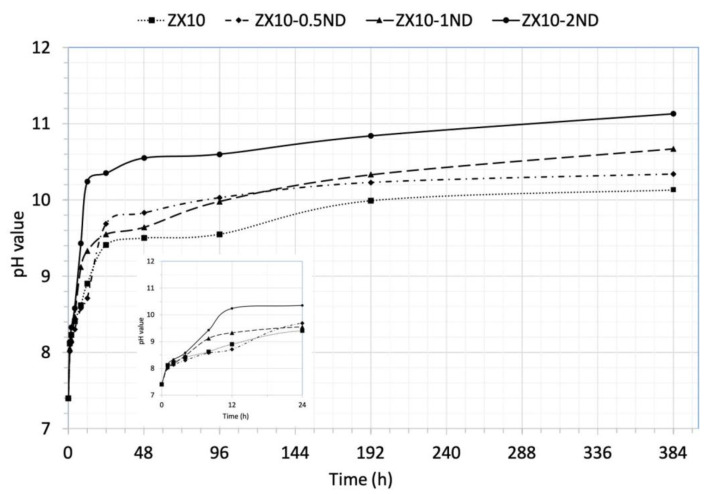
The pH value of HBSS as a function of immersion time for ZX10 alloy and ZX10-0.5ND, ZX10-1ND, and ZX10-2ND.

Figure 10 exhibits the changes in the pH value of matrix alloy and of nanocomposites in the HBSS during immersion depending on time. It can be seen in Figure 10 that the pH value of the solution increased rapidly in all samples during the first 3 h of immersion, and then it increased more slowly and more stably. This trend can be ascribed to the formation of Mg (OH)_2_ on the specimens’ surface. The pH value of the solution was found to be 10.13 for the ZX10 alloy. Furthermore, the pH values of the solution for all of the nanocomposites were found to be higher than that of the alloy, and they were determined to be 10.34, 10.67, and 11.13 for ZX10-0.5ND, ZX10-1ND, and ZX10-2ND, respectively. 

**Figure 11:**
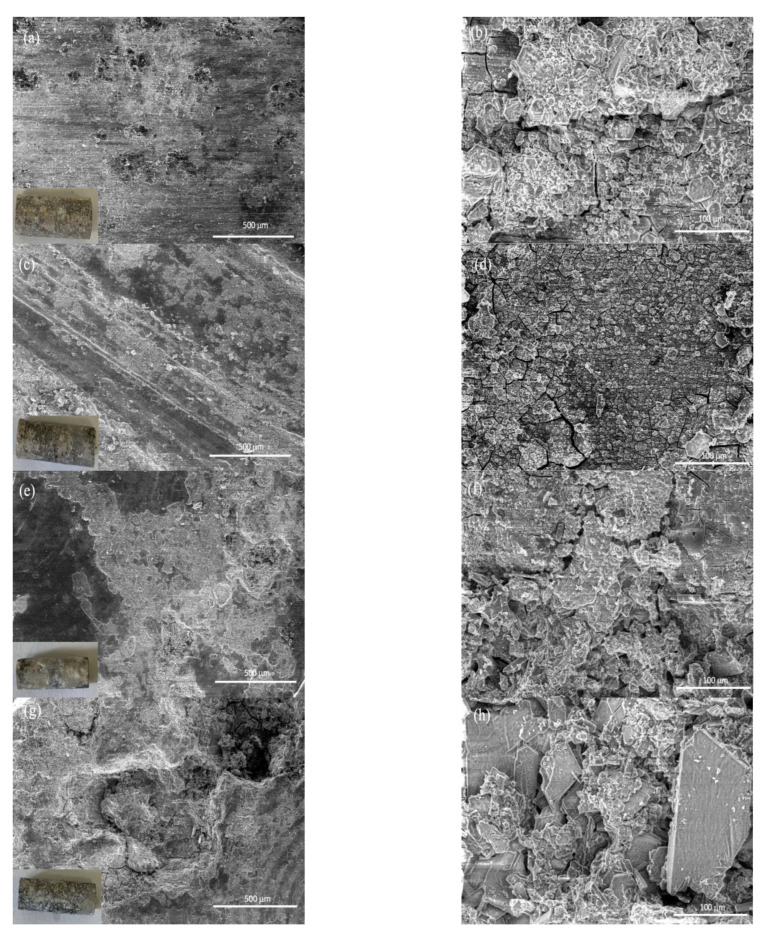
The surface morphologies of samples immersed in HBSS for 384 h: (**a**,**b**) ZX10 alloy, (**c**,**d**) ZX10-0.5ND, (**e**,**f**) ZX10-1ND, and (**g**,**h**) ZX10-2ND.

To better understand the corrosion process of each sample, the surface morphologies of samples immersed in HBSS for 384 h were analyzed by scanning electron microscopy and shown in Figure 11. The literature declared that the corrosion of magnesium is mainly localized corrosion caused by the formation of pits and cracks [51]. As shown in Figure 11, corrosion cracks and pitting were observed on both the alloy and composite surface, and even severe corrosion holes were found on the ZX10-2ND surface.

**Figure 12:**
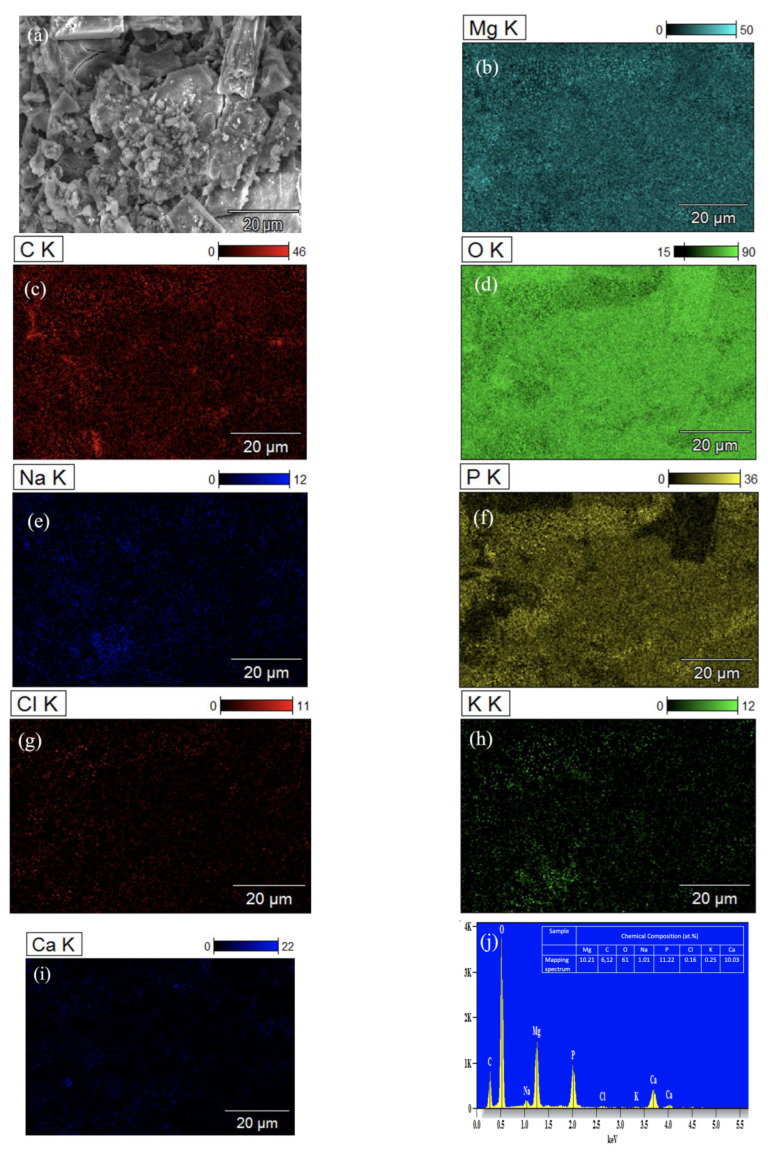
EDS analysis of ZX10-2ND: (**a**) image; (**b**) Mg; (**c**) C; (**d**) O; (**e**) Na; (**f**) P; (**g**) Cl; (**h**) K; (**i**) Ca; and (**j**) spectrum.

Figure 12 shows the analysis of corrosion products formed on the surface of the ZX10-2ND with EDS mapping after 384 h of immersion. It can be observed that the surface corrosion products were mainly the content of O, P, Ca, and Mg, which indicates the existence of amorphous corrosion products such as (Ca_0.86_Mg_0.14_)_10_(PO_4_)_6_(OH)_2_ due to the content of the HBSS solution [17].

The reason for the increase in pH value may be the increase in galvanic corrosion among the nanodiamonds and α-Mg matrix in parallel to the increase in the amount of added nanodiamonds because of an increase in the area ratio of cathode-to-anode, thus forming more galvanic couples. Similar effects of nano carbon in the Mg matrix have been identified by some studies [24,46,47]. For instance, Fukuda et al. [46] reported the formation of galvanic cells between the CNTs and α-Mg matrix of a AZ31B alloy. In another study by Aung et al. [47], it was reported that the corrosion rate increased in the Mg/CNTs composites because of microgalvanic action among the α-Mg matrix and CNTs.

### 3.4. Potentiodynamic Polarization Test

**Figure 13:**
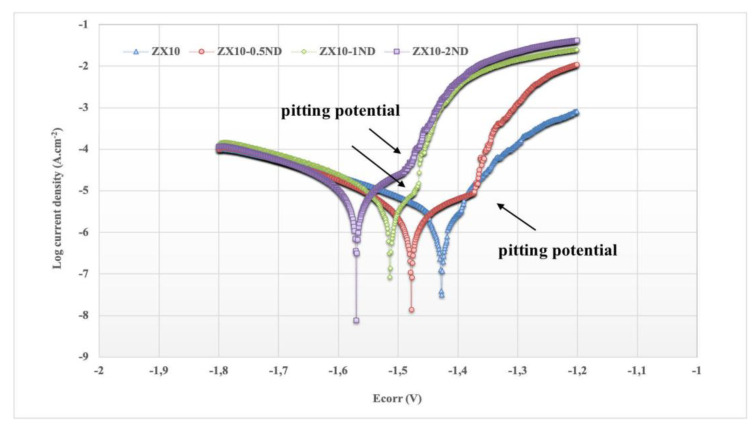
Potentiodyamic polarization curves of ZX10 alloy and nanocomposites in HBSS.

The polarization curves of the ZX10 alloy and of the nanocomposites in the HBSS are shown in Figure 13. There are two parts in the polarization curve of each sample as an anodic reaction (right branch) ascribed to the dissolution of Mg leading to formation of Mg^+2^ according to Equation (1), and as a cathodic reaction (left branch) indicating the hydrogen evolution reaction according to Equation (2). The corrosion potential (E_corr_) and corrosion current density (I_corr_) of the samples, calculated by the polarization curves in Figure 13, are listed in Table 2.

It can be observed from Figure 13 and Table 2 that the value of the corrosion potential of the ZX10 alloy was found to be −1.427 V. The values of the corrosion potentials for ZX10-0.5ND, ZX10-1ND, and ZX10-2ND tended towards the negative direction by the addition of nanodiamond, and they were found to be −1.478, −1.514, and −1.570 V for ZX10-0.5ND, ZX10-1ND, and ZX10-2ND, respectively. In addition, the corrosion current densities of the samples obtained were 1.75, 2.01, 3.42, and 5.28 µA.cm^−2^, respectively. Furthermore, it can be seen in Figure 13 that the pitting potential of the nanocomposites polarization curves, which implies the tendency for localized corrosion, is more negative than that of the ZX10 alloy. Therefore, localized corrosion can occur more easily in the nanocomposites. Besides, the polarization curves show that an acceleration of the cathodic hydrogen evolution kinetics is observed when nanodiamond particles are added to the ZX10 alloy. However, the kinetics of the anodic reaction are not considerably affected by the existence of the nanodiamond particles. Therefore, the influence of the nanodiamond particles on the electrochemical behavior of the Mg-Zn-Ca alloy is similar to the well-known effects of impurities such as Fe or Cu [51]. 

**Figure 14:**
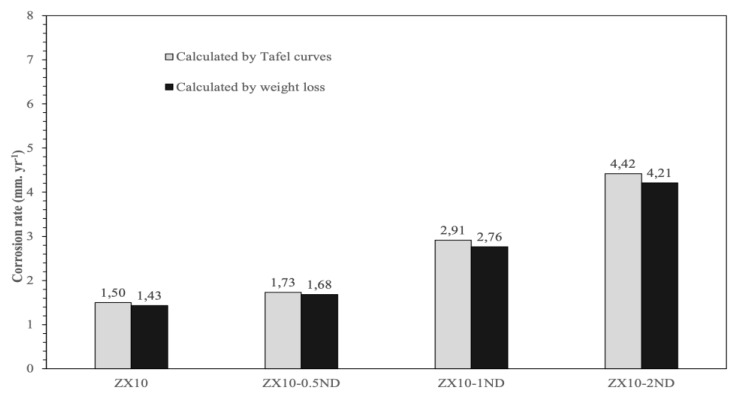
The average corrosion rates of the ZX10 alloy and ZX10-0.5ND, ZX10-1ND, and ZX10-2ND nanocomposites.

The average corrosion rates of the samples were calculated by weight loss and by Tafel curves, and they are represented in Figure 14. Based on the weight loss and Tafel curves, the ZX10 alloy exhibited the lowest corrosion rate by 1.43 and 1.5 mm.yr^−1^, while the corrosion rates of ZX10-0.5ND, ZX10-1ND, and ZX10-2ND were 1.68, 1.73 mm.yr^−1^, 2.76, 2.91 mm.yr^−1^, and 4.21, 4.42 mm.yr^−1^, respectively. It can clearly be seen that the corrosion rate of the alloy increased by the addition of nanodiamonds, although a smaller grain size was obtained. Consequently, the best mechanical properties among the nanocomposites were obtained with the addition of wt.% 0.5 ND, although the corrosion rate of the ZX10 alloy slightly increased.

## 4. Conclusions

The effect of nanodiamond particles on the microstructure, mechanical properties, and corrosion behavior of the ZX10 alloy was investigated. The following results can be surmised:

1. The ZX10 alloy and nanocomposites mainly consist of the α-Mg and Ca_2_Mg_6_Zn_3_ intermetallic phases. In addition to this, the diamond peak was detected in the ZX10-2ND nanocomposite.
2. The microstructure observation exhibited grain refinement of the ZX10 alloy with the addition of nanodiamond particles. Grain refinement may have been triggered by the action of the nanodiamonds as crystallization nuclei during solidification. This is a positive effect in terms of the use of the material as an implant material as it has a strengthening effect. This is visible in the increasing tendency of the yield strength with increasing nanodiamond content.
3. The nano hardness and elastic modulus values of the ZX10 alloy increased with the increasing the number of added nanodiamond particles.
4. The corrosion rate of the ZX10 alloy increased drastically with an increase in the wt.% nanodiamond particles. Only 0.5% nanodiamonds leads to a corrosion rate that seems to be acceptable for a degradable implant. The nanocomposites with 1 and 2% nanodiamonds already have a corrosion rate that is too high.

## Figures and Tables

**Table 1:** Nominal and actual composition of Mg-Zn-Ca alloy using X-ray fluorescence (Bruker S8 Tiger, Billerica, MA, USA) analysis.

Sample	Magnesium (wt.%)	Zinc (wt.%)	Calcium (wt.%)
Mg-1Zn-0.6Ca	98.37	1.07	0.56

**Table 2:** Electrochemical parameters and corrosion rates of samples in HBSS obtained by polarization test.

Sample	E_corr_ (V)	I_corr_ (µA.cm^−2^)	Corrosion Rate (mm/year)
ZX10	−1.427	1.75	1.5
ZX10-0.5ND	−1.478	2.01	1.73
ZX10-1ND	−1.514	3.42	2.91
ZX10-2ND	−1.570	5.28	4.42
